# A history of metaphorical brain talk in psychiatry

**DOI:** 10.1038/s41380-025-03053-6

**Published:** 2025-05-13

**Authors:** Kenneth S. Kendler

**Affiliations:** 1 Virginia Institute for Psychiatric and Behavioral Genetics, Virginia Commonwealth University, Richmond, VA USA; 2 Department of Psychiatry, Virginia Commonwealth University, Richmond, VA USA

**Keywords:** Neuroscience, Depression

## Abstract

From the very beginnings of our field in the late 18th century, psychiatrists have engaged, often extensively, in “metaphorical brain talk” – rephrasing descriptions of mental processes in unconfirmed brain metaphors (e.g., “diseased working of the brain convolutions”). In the late 19th century, Kraepelin criticized the later developments of such approaches, termed “brain mythology” by the philosopher/psychiatrist Jaspers in 1913. In this essay, I review the history, meaning, and significance of this phenomenon and reach four conclusions. First, this trend has continued to the present day in metaphors such as the “broken brain” and the use of simplistic and empirically poorly supported explanations of psychiatric illness, such as depression being “due to an imbalance of serotonin in the brain.” Second, our language stems from the tension in our profession that seeks to be a part of medicine yet declares our main focus as treatment of the mental. We feel more comfortable with the reductionist approach of brain metaphors, which, even though at times self-deceptive, reinforce our commitment to and membership in a brain-based medical specialty. Third, metaphorical brain talk can also be seen as the “promissory note” of our profession, a pledge that the day will come when we can indeed explain accurately to ourselves and to our patients the brain basis of the psychiatric disorders from which they suffer. Finally, moving away from metaphorical brain talk would reflect an increasing maturity of both the research and clinical aspects of our profession.

Psychiatry emerged as a medical specialty between 1780 and 1830 committed both to the care of the mentally ill and to the brain as the seat of these disturbances [1]. This created an inherent tension, because for all of our history up to the present day, with rare exceptions, such as general paresis of the insane, we have had little insight into exactly how disturbances in the brain cause psychiatric disorders. This is not the case with most other organ-based medical specialties, like obstetrics and ophthalmology, where organ-specific pathologies could be related to many observed clinical syndromes. This article seeks to describe how the tension between the mental, clinical phenomenon we treat and our commitment to brain-based explanations as a medical specialty have played out over the history of our discipline.

We proceed historically and focus on a concept I call “metaphorical brain talk,” defined as *describing the disturbed mental processes in psychiatric illness in terms of brain function in ways that appear to be explanatory but actually have little to no explanatory power*. I will work my way through (i) 19th century asylum psychiatry, (ii) the rise and then decline of the first biological psychiatric revolution in the 1860s–70s and 1880–1910 respectively, (iii) meet the key figure of Meynert and the application to his work of the concept of “brain mythology” by Karl Jaspers, (iv) trace a few examples of metaphorical brain talk into the 20th century, and (v) review a more positive view of brain talk – as instantiating a promissory note of our profession to one day clarify the brain mechanisms underlying psychiatric illness. I conclude by summarizing the significance of this phenomenon, postulating that it addresses a foundational conflict in our medical profession of psychiatry between our commitment to the study and treatment of the disturbed mental phenomenon and our identification with the brain as our organ of interest.

## Metaphorical brain talk in asylum psychiatry 1780–1900

Here are twelve examples of metaphorical brain talk sampled from over a century from physicians who cared for and wrote, largely in asylums, about the nature and origins of what they would have called madness or insanity. Italics are added throughout this essay for emphasis:

Cullen 1784 – Delusions, he suggests “may depend … upon some *inequality in the excitement of the brain* …[for] the proper exercise of our intellectual functions, the excitement must be complete, and equal in every part of the brain …. so, if any part of the brain is not excited, or not excitable, that recollection cannot properly take place, while at the same time *other parts of the brain, more excited and excitable, may give false perceptions, associations, and judgments”* [2] pp. 130–131.

Hartley 1834 – Who, in describing a potential mechanism for delusions, writes: “thus suppose a person, whose nervous system is disordered, to turn his thoughts accidentally to some barely possible good or evil. If the nervous disorder falls in with this, *it increases the vibrations belonging to its ideas so much, as to give it a reality*…” [3] p. 252.

Laycock 1845 – He explains the origin of obsessions “..by which is termed the association of ideas, *the morbid action of vesicular neurine* be brought within the current of his thought, he becomes utterly powerless to resist it…” [4]. [Neurine, an alkaloid found in egg yolk, brain, bile and decaying flesh arises from the putrefaction of biological tissues and is a syrupy liquid with a fishy odor.]

Monro 1851 – He proposes a theory of insanity: “1. That it is an affection consequent on depressed vitality [which] manifest itself with *peculiar and specific force in the cerebral masses*, owing to a congenital, and frequently hereditary, *tendency in the brain thus to succumb when oppressed by any exciting cause*. 2. That when the cerebral masses are suffering from this condition of *depressed vitality, they lose that static equilibrium of the nervous energies* which we call tone” [5] p. 76.

Dickson 1874 – The characteristic of some forms of insanity is excitement and vividness of impression, *but this excitement and vividness always emanate from one portion or spot of the brain* which pays out its functional activity rapidly and unchecked or uncorrected by the [brain] portions whose functions are arrested or in abeyance [6] p. 11.

Ball and Ritti 1881 – the cells of the cortex we are told are the organ of intelligence; it is therefore right that *to their disorder, whether anatomical or physiological, the production of delusions should be attributed* [7] p. 337.

Stearns 1883 – The Professor takes notice of two states of the brain; the one he terms excitement—the other collapse. Collapse may be defined *a morbid diminution of the tone of the brain, and of the motion of the nervous fluid*. The term excitement must be obvious to everyone … sometimes a *collapse of one part of the brain interrupts the communication of the due excitement of the whole, and thus induces delirium [delusions]* [8] p. 6–8.

Savage 1884 – With melancholia we meet with a slowing of all vital processes. What the pathological basis of melancholia may be one cannot at present tell. It however seems, in most cases, that *it must be associated with impaired nutrition of the nervous centres and the conducting system* [9] p. 130.

Clouston 1892 – An “insane delusion” may therefore be defined to be “a belief in something that would be incredible to people of the same class, education, or race as the person who expresses it, the belief persisting in spite of proof to the contrary, this resulting from *diseased working of the brain convolutions*” [10] p. 244.

Clark 1895 – The factor which is inherited [in severe mental illness] cannot be insanity per se, but may be *an instability or disordered arrangement of nerve tissue* [11] p. 238.

Maudsley 1895 – In a description of melancholia, he writes “The probable pathological condition of things is an exorbitant and predominant, almost exclusive, *activity of certain brain-tracts charged with sad feeling* not unlike the sort of activity which has motor issue otherwise in spasm or convulsion of muscles” [12] p. 195.

Kellogg 1897 – In explaining hallucinations, he write that “The main pathogenic fact is that *the sensory image is aroused in the higher brain centres with such unnatural force* as to be projected outwardly as an objective reality” [13] p. 152.

The goal of each of these examples of brain talk is to explain insanity itself or some more specific features like delusions or melancholia in terms of brain function or structure. But the descriptions are non-specific and/or metaphorical and lacked substantial biological meaning. We find general constructs such as “inequality of excitement,” “morbid action of vesicular neurine,” “diminution of the tone of the brain,” “impaired nutrition of the nervous centres,” “diseased workings of the brain convolutions,” and “disordered arrangement of nerve tissue.” Nearly all these observations could have been stated in mental language, but were not.

## Brain talk and the first biological revolution in psychiatry 1870s–1880s

The first biological revolution in psychiatry, the turn to neuroanatomy and neuropathology, was largely stimulated by Wilhelm Griesinger (1817–1868) [14, 15], the first Professor of Psychiatry in German speaking Europe and the authors of an influential textbook of psychiatry [16–18]. He established the important journal *Archives of Psychiatry and Nervous Diseases* in 1868 and in his preface to the first issue wrote:

Psychiatry has undergone a transformation in its relation to the rest of medicine.… This transformation rests principally on the realization that patients with so-called ‘mental illnesses’ are really individuals with illnesses of the nerves and brain [14] p. 76.

Eric Engstrom, a leading historian of German Psychiatry, captures the critical change that Griesinger initiated in German psychiatry:

During the 1850s and 1860s, his textbook had served as a powerful catalyst to an entire generation of students … who wholeheartedly adopted Griesinger’s conviction that “mental disease were brain diseases” and who went about applying anatomic and physiologic methods of inquiry” [15] p. 90.

His students, particularly Westphal, Meynert, and Wernicke, became the first generation of academic psychiatrists in the world, pursuing biological/neuropathological research careers outside of asylums. Griesinger strongly advocated for the establishment of psychiatric autopsies as the key research method. Disciplined science applied to the brains of the diseased mentally ill would, he asserted, reveal an anatomical diagnosis, the true essence of mental illness [15]. In the increasing number of university-based departments of psychiatry in German-speaking Europe established in this era, the leading scientific paradigm, the high prestige research programs, were in neuroanatomy and neuropathology.

The excitement of this era in psychiatry was well captured by Otto Binswanger in 1892:

Under the influence of the tremendous progress of medical science in anatomy and physiology [and] … the increase in the use of the microscope… a degree of disdain for imponderable psychic influences became the norm. This progress literally intoxicated the heads of many; in heated efforts to derive the cause … of all pathological life processes from the fundamental precepts of biological [and anatomical] work… [19] p. 53.

But the excitement was short lived. The hope that gross and/or microscopic examination of the brains of the mentally ill would yield insights into etiology of the classical psychiatric disorders was not fulfilled. But something else happened in the wake of these failures, a proliferation of more elaborate metaphorical brain talk.

## The reaction to this “Revolution”

In a section of his history of this era, roughly 1880–1910, entitled “Neuropathology in Retreat,” Engstrom begins:

At the close of the century, a number of critics of neuropathology emerged within the ranks of the profession to add their voice to [other] longstanding … objections … the fruits of anatomical research turned out to be less than originally expected…. The potential of anatomical research had been decidedly overestimated and … researchers had fallen victim to speculative interpretations of their laboratory results….Research had stagnated in what had become an era of abstract theories and schematic models of brain function in which researchers appears to take little notice of alternative theories or of contradictory evidence [15] p. 123.

One early critique of this new neuropathological biological psychiatry was provided by a 31-year-old Emil Kraepelin. In the inaugural lecture for his first professorship at the University of Dorpat in 1887 [20], he summarized the current progress in psychiatry and provided a strong critique of the biological theorizing then occurring from prominent neuroanatomical psychiatric researchers, especially Meynert [21], who was then working at the University of Vienna. He began by noting that “the impossibility of a satisfactory solution [to the mind-body problem] … has led to numerous attempts to bridge the gap separating events of the body and mind *by means of airy constructions of speculative fantasy*” p. 351. This focus on highly speculative brain-based theories “… explain the strange fact that psychiatry still finds itself in an era of fruitless hypotheses and theories – an era long surpassed by the rest of medical science…” [21] p. 351.

Then, he directly confronts the reductionist biological theories of Meynert, in which he defined his science as a clinical science of the diseases of the anterior brain, its structure, capabilities and nutriments. In the field of cerebral anatomy*, in spite of wholly inadequate factual grounding*, he ingeniously understood and described the general blueprint of the organs of the central nervous system [21] p. 353.

Meynert, according to Kraepelin, took the concept of localization of cerebral functions to implausible extremes.

He does not even shy away from the shocking implication … that *every cell … must be viewed as the seat of a specific and discrete idea*; and that the associative linkage of that idea with other mental elements is mediated through connective pathways that he calls associative strands. The second key idea behind Meynert’s theories is taken from neural physiology and is expressed in the terms ‘stimulation’ and ‘inhibition.’ As soon as each individual mental function has been associated with the various parts of the brain, then naturally *it suffices to posit an inhibition here and a stimulation there in order to explain the most diverse combinations of phenomena* [21] p. 353.

Kraepelin continues to his key point, noting that some of Meynert’s anatomical claims had already been contradicted by recent research.

It is possible that specific aspects of this system will establish themselves as an enduring part of our science. But if that is to be the case, then it will happen only after much long, arduous and detailed work has been completed… Only time will tell whether, as in the case of anatomy, the basic idea represents an intuitive knowledge of reality and proves to be true. *But it is dangerous – and Meynert’s school has not entirely escaped the ruinous consequences – to want immediately to move into and live in Meynert’s airily-constructed house, before its foundation has been securely laid and hard work has tightened up, one by one, the loose joints of his theory*… [21] p. 354.

He then generalizes his criticism to a number of the highly speculative reductionist physiological theories postulated during this time period:

While Meynert’s constructions are based on assumptions that appear to be open to empirical inquiry at some point in time, *there is no shortage of* [other] *theories built on ground that will probably forever remain in the realm of hypothesis*. Recall for example the attempts to transcribe simple [mental] processes into the language of psychophysics, or the efforts to trace the origins of mental disorder back to the gradual death of the core neural material, or finally the ingenious essays that plumb the depths of molecular mechanics in order to arrange the physiological processes that supposedly occur in the disordered brain in terms of the interaction of elementary forces, the exchange of kinetic into potential energy and vice versa, or even the movement of atoms. *The impartial observer will barely be able to contain his astonishment when he sees how, in the very medical discipline most lacking in factual and scientifically useful empirical evidence, there exists a flourishing tendency to extend theory all the way back to the most primal state of the phenomena* [21] p. 354.

## More on Meynert

As a major figure in the history of neuroanatomy and chair of the department of psychiatry in Vienna from 1873–1892, Theodor Meynert (1833–1892) is worthy of more attention as he typifies in this historical era, the excess exuberance of metaphorical brain talk. Here is an extended quote from a careful review of his career and research by the eminent Austrian neurologist and historian – Franz Seitelberger [22]:

Meynert carried his morphological research to its attainable limits and then transgressed them by hypotheses … [His] model, while partly based on empirical morphological findings, also involved *some deductive speculative thinking, devoid of proof, yet fused into a coherent whole*. The structural element of the brain, the nerve cell is, in Meynert’s view, endowed with a ‘soul’… Meynert conceives of the function of the *cerebral hemispheres as analogous to “colonies of living beings, capable of consciousness, connected with each other with feelings threads … and controlling their image of the world*” [23] p. 269.

We will not detail with other aspects of his system except to note that some tracts in the brain reflected “unconscious animal life,” while others, especially the cortex, constituted the “ego-forming functional center of the brain.”

His developed distinct theories of psychotic and mood-based psychopathologies, with the former arising from

Disturbed cooperation between the various parts of the brain, a disturbance of association coordination often caused by “functional disturbances of the social nature of the brain” [23] p. 270.

By contrast, his theory of affective disturbances, was an elaboration of a very old view, based in humoral medicine, of excess or deficient blood flow. Seitelberger summarizes his theory:

They combined the assumption of an aggressive nature of neuronal activity with the functional role of the cerebral blood flow and the antagonism between cortex and subcortical centers…. The *feeling of unhappiness is linked with an inhibition or suppression of the neuronal activity* … and corresponding reduction in blood flow. Affects and therefore manifestations of the nutritional state of the cortical neurons [23] pp. 270–271.

Another psychiatric historian, Janzarik, summarized Meynert’s neuropathological work as follows

Paradigmatic for the spirit of the time is the highly speculative fusion of psychopathological findings with anatomical findings and pathogenetic claims, which in Meynert, following an old tradition, relate in particular to vascular-dependent differences in brain nutrition [24] pp. 598–599.

Meynert’s own student, Auguste Forel, called his brain pathways “fantastical constructions” [25] p. 177. We conclude with one further critique from Phelps:

What about Meynert’s work made it cogent and compelling? What about it seemed capable of dispelling the mystery of the nervous system and dispensing with the unique inwardness of the mind? Part of the answer … lies with Meynert’s images of the brain’s material and the collocation of its fleshy, fibrous inner stuff with the interiority of the mind… By delineating various ‘fibre-systems’ in the brain and nerves, then deducing their different functions on the basis of their winding ‘pathways’, Meynert elaborated new shapes and textures inside the brain and by doing so, he elaborated … [and] fleshed-out functions of the mind… *But even as he imagined these fibres as pathways or tracks inside the brain, he co-extended them and co-located them as pathways somehow equally inside the mind* [26]. P. 389–390

His success, Phelps suggests, was due to his ability “to connect what he saw inside the brain with an image of what he described as ‘inside’ the mind [by] … *his combination of material and metaphorical techniques*” [26] pp. 394.

## Metaphorical brain talk in the 20th century

We first turn to Adolf Meyer, the most influential psychiatrist in the US over the first 3rd of the 20th century [27]. It should be recalled that until World War II, American psychiatry was a rather small profession, largely composed of superintendents of mental hospitals who largely had a biologically orientation to their work. In 1907, while the director of the New York Psychiatric Institute, Meyer wrote about his concerns of the narrow views that US physicians would typically take in their approach to psychiatric illness that likely reflected his views about the excesses of earlier authors like Meynert:

Instead of analyzing the facts in an unbiased way and using the great extension of our experience with mental efforts to get square with things … they pass at once to a one-sided consideration of the extra-psychological components of the situation, abandon the ground of controllable observation, *translate what they see into a jargon of wholly uncontrollable brain-mythology, and all that with the conviction that this is the only admissible and scientific way* [28] p. 172.

Next, we examine a text on a similar theme from a quite different source - the psychiatrist-philosopher Karl Jaspers. In his the introduction to the first (1913) edition of *General Psychopathology* [29], he writes:

The still widespread “somatic prejudice” is: everything mental cannot be examined as such, it is merely subjective. If it is to be discussed scientifically, it must be presented anatomically, physically, as a physical function; for this it is better to have a preliminary anatomical construction, which is considered heuristic, than a direct psychological investigation*. Such anatomical constructions are quite fantastic (Meynert…) and are rightly called “brain mythologies.”* Things that have no connection to one another, such as cortical cells and memory images, brain fibers and psychological associations, are brought together. *There is also no basis for these mythologies insofar as not a single specific brain process is known that could be assigned to a specific mental process as a direct parallel phenomenon* [29] p. 8 (KSK translation).

We might think that recent scientific developments over the rest of the 20th century eliminated the need for metaphorical brain talk. We provide three examples suggesting that this is not the case.

First, in a series of articles published over several decades, the distinguished psychologist Paul Meehl proposed a cognitively and psychometrically sophisticated genetic single-locus model for schizophrenia spectrum disorders. A core part of this theory was equating cognitive and neurobiological parts of his theory, as expressed here in 1962:

The cognitive slippage is here conceived as a direct molar consequence of *synaptic slippage*, potentiated by the disruptive effects of aversive control and inadequate development of interpersonal communication sets [30] p. 834.

His phrase, “synaptic slippage,” has commonly been repeated in the subsequent literature [31, 32].

Second, in 1985, a leading biological psychiatrist, Nancy Andreasen, published a widely-cited book whose title was a paradigmatic example of metaphorical brain talk: *The Broken Brain* [33]. She writes, for example, that recent advances in research have “taught us that many forms of mental illness are due to abnormalities in brain structure or chemistry. Psychiatry is moving from the study of the “troubled mind” to the “broken brain” [33] p. VIII. In a later section, she describes, using broad metaphors, the kinds of brain abnormalities that occur in psychiatric disorders.

The various forms of mental illness are due to many different types of brain abnormalities … sometimes the fault maybe in the pattern of the wiring or circuitry, sometimes in the command centers, and sometimes in the way messages move along the wires” [33] p. 221.

Third, in an important development in the history of neuroscience, in the early 1960s, cell bodies and neuronal pathways of the putative monoamine neurotransmitters dopamine, norepinephrine, and serotonin were demonstrated in mammalian brains [34–36]. Within a few years, prominent psychiatric researchers proposed that abnormalities of function in these neurotransmitters were the major cause of three of the most important of psychiatric disorders: schizophrenia, mania and depression [37–40]. I suggest that these theories reflect, in more subtle ways than prior examples, metaphorical brain talk. Or, perhaps these monoamine hypotheses could be seen as sitting somewhere on a continuum of naively enthusiastic scientific theories and metaphorical brain talk. They were grounded in solid basic neuroscience, and had support from pharmacologic studies of mechanisms of action of antipsychotic and antidepressant medication [41]. However, trying to clarify disease etiology through the mechanism of action of pharmacologic treatments is deeply problematic as illustrated by the now common phrase: “headache is not an aspirin-deficiency disease” [42]. Given the more than 100 neurotransmitters in the mammalian brain, the plausibility that dysfunctions in the first three to be traced in the brain caused the major psychiatric disorders strains to the breaking point any sense of credulity. Furthermore, these theories, for example, the serotonin hypothesis of depression [43–47], have not fared well over time. Large-sample, genome-wide association studies are now available on all three disorders, and none support a major role for genetic variants involved in the dopamine, norepinephrine, and serotonin systems for, respectively, schizophrenia [48], bipolar illness [49], and depression [50]. A recent widely-cited umbrella review concludes, “The main areas of serotonin research provide no consistent evidence of there being an association between serotonin and depression, and no support for the hypothesis that depression is caused by lowered serotonin activity or concentrations” [46] p. 3243. While there remains substantial controversy about the precise etiological relationship between serotonin and depression [43, 51, 52], I do not seek to deny that serotonin may play some role in the complex pathophysiology of this syndrome. Rather, I suggest, less controversially, I hope, that the grand monocausal theory of depression resulting primarily from serotonin dysfunction (as I have argued earlier, regarding the dopamine hypothesis of schizophrenia [42]) is almost certainly false.

Importantly, these monoamine theories have had impacts outside of our research world. I recently heard the following story from friend of a friend, who knew I was a psychiatrist:

I was feeling really down and my family doctor referred me to a local psychiatrist, Dr. C. We talked for 30 min. He said I had what he called “major depression” and this was due to an imbalance in my brain serotonin. He said I should take the medicine he prescribed and it would correct that imbalance. Three weeks later I was feeling a lot better. I was really impressed.

While certainly more false than true, this example of metaphorical brain talk remains a common story in psychiatric culture. Some of us tell stories like this to our patients, in part because it may make them feel better and also perhaps because it is a story we like to tell. Metaphorical brain talk can be popular.

While my focus in this essay is the internal history of the psychiatric profession, there remains one elephant in the room that needs brief attention. When psychiatric research came to seek external financial support, simplistic metaphorical brain talk often appealed to prospective funders. This continues to the present day. Even more powerfully, advertising of psychopharmacological drugs, rising to prominence in the last third of the 20th century, often revolved centrally around metaphorical brain talk. While we like to claim that such advertising has no effect on us, this is naïve. An empirical review of this question, while far outside my remit, would, I suspect, find such advertisements have significantly increased the modern popularity of metaphorical brain.

## Why brain talk?

Metaphorical brain talk has arisen out of a foundational feature of our profession. Psychiatry began and remains a profession that treats disorders whose major clinical manifestations are in mental space – symptoms – as well as resulting signs and disturbed behaviors. But, we are also a medical specialty and consider our association to medicine central to our mission and professional identity. Most other medical specialists have organs of special focus – ophthalmology the eye, cardiologists the heart, gynecologists the uterus and ovaries. In its first decades in the late 18th and early 19th centuries, psychiatry, for quite logical reasons, chose the brain [1]. But, some 80 years later, neurology began to develop and took with it nearly all the diseases where, given the methods of gross and histological pathology then available, one could track those disorders back to definable brain or nervous system lesions. Ever since, our relationship with the brain has been an ambivalent one, the reasons for which can be simply explained. The indirect evidence that the symptoms of our disorders are instantiated in brains is overwhelming and new genetic studies are providing a further sound footing to this long widely accepted belief [53]. However, despite decades of research, and tremendous progress in basic neuroscience, imaging technologies, and molecular genetics, we still have no good idea exactly how psychiatric disorders emerge from disturbances in brain function. This is an uncomfortable position for our field to be in. Metaphorical brain talk has, for now more than two centuries, been one way to patch over this discomfort.

The medical historian Rosenberg can help us here, first in a “status” report on our discipline:

Since its origins as a specialty in the 19th century, psychiatry has … suffered from a recurrent status anxiety—one might call it procedure envy, or organic inferiority. Psychiatry has been chronically sensitive to its inability to call upon a repertoire of tightly bounded, seemingly objective, and generally agreed-upon diagnostic categories based firmly on biopathological mechanisms … Psychiatry remains the legatee of the emotional, the behavioral, and the imperfectly understood. In this sense it has been a poor relation of its specialist peers in surgery and internal medicine [54] p. 124.

Second, in reviewing the history of 19th century medicine and the success of its system of primitive etiologic theories, he makes the following point relevant to my argument

…the [medical] system provided a rationalistic framework in which the physician could at once reassure the patient and legitimate his own ministrations…. The physician’s own self-image and his social plausibility depended on the creation of a shared faith – a conspiracy to believe - in his ability to understand and rationally manipulate the elements in this speculative system [55] P. 489.

Even though not true in any substantive scientific sense, since the beginning of our discipline, metaphorical brain talk has, as part of a professional “conspiracy to believe,” helped our own self-image as a “poor relation” in medicine and given us plausible metaphors to communicate about the disease we treat with ourselves and with our patients. This conspiracy, I suggest, was further reinforced through the large advertising budgets of modern drug companies.

## Another take on metaphorical brain talk

Having read and pondered many of the 19th century examples from asylum psychiatrists of metaphorical brain talk, and the more elaborate high speculative system of Meynert, I realized that there is a more positive view one might take of these efforts. It complements the more critical perspective outline above. These examples of brain talk also speak of the deep desire of psychiatrists to truly understand the brain basis of mental illness. For many of them, their constant pattern of framing mental descriptions of psychiatric illness in brain metaphors likely bespeaks their wish this could one day be done in earnest. Their use of brain talk was indirectly an expression of a promissory note for the future, a long-term aspiration to one day be able to both be a clinician, familiar with the description and treatment of the deeply disturbing mental symptoms of their patients, and, like their colleagues in other branches of medicine, have the ability to identify, by clinical signs, laboratory or radiological tests, or even biopsy, a definitive underlying pathophysiology in the brain.

I present only one example of this phenomenon from the influential psychiatrist Kurt Schneider (1887–1967). In the fifth edition of his short textbook *Clinical Psychopathology* [56], in his section on classification, he describes two groups of disorders – those termed “abnormal variations of psychic life” and the other “effects of Illness.” All the latter have clear somatic etiologies (e.g., general paresis of the insane, cerebral damage), except schizophrenia and what Schneider called “cyclothymia,” equivalent to Kraepelin’s manic-depressive illness. Why were they classified differently from the other psychiatric syndromes? He explains:

There is no question at this point whether some morbid condition does in fact underlie these psychopathological forms … Here we are firmly postulating that cyclothymia and schizophrenia are psychopathologic symptoms of some unknown illness. If “postulate” seems too strong, we can say “working hypothesis” instead [56] p. 5.

In the absence of clear evidence, Schneider was willing to take a “promissory note” on the hypothesis that Kraepelin’s two major disorders of schizophrenia and manic-depressive illness were indeed the result of some as yet “unknown illness.” Perhaps Schneider was expressing overtly what many of the earlier brain talkers were feeling covertly – “there are real diseases down there – give us time, and we will find them.”

## Brain talk and reductionism

Metaphorical brain talk is the soft underbelly of the reductionist agenda of modern science, which, since the dawn of the Enlightenment, has sought to explain the complex phenomena we confront in our biological and physical environment on the basis of simpler physical constituents [57]. This approach has been spectacularly successful in physics, chemistry, and molecular biology, as driven by highly quantitative, replicable science. Its application to the world of the mental has been less successful and more problematic. Metaphorical psychiatric brain talk adopted the language of reductionism without its empirical scientific underpinning.

To be clear, my criticism of metaphorical psychiatric brain talk is not a critique of the rigorous scientific reductive agenda in modern psychiatric research that seeks to understand the etiology of psychiatric illness. Indeed, I have spent most of my career in the study of psychiatric genetics, clearly, at least in part, a reductive research project. My concern is rather the degree to which the profession of psychiatry, over its long history, has impoverished its conceptual foundations by a strong brain-focused bias in how we talk, and, more importantly, think about mental disorders. We are at risk of underappreciating efforts to understand the first-person experiences of our patients. If our reductionist neuroscience efforts are to succeed, we will have to be able to link our scientific explanations with incisive understanding of the states of mental illness we treat. We could then provide our patients not with empty, metaphorical brain-talk, but a real explanation of the ways in which brain dysfunctions produce their experiences. This will, in turn, hopefully expand our ability to emphasize with their symptoms in the process of “explanation-aided understanding” [58].

## So what?

We have a very imperfect sense of the brain-based disturbances that predispose our disorders. We should not be ashamed of this. We have been working hard for a very long time on a set of problems of extraordinary complexity. Talking about our profession and our disorders to ourselves, our colleagues and patients using metaphorical brain talk is scientifically immature and ultimately disrespectful to our patients. Unless we know otherwise, we should assume that our patients want us to “Tell it like it is, Doc,” even when that means we cannot tell pretty stories about serotonin imbalances. When we describe the suffering of our patients, we don’t have to “dress up” the descriptions of their mental anguish with problematic brain metaphors. We should tell them what we know, with all the uncertainty. We should take pride in being the only specialists in medicine that have chosen to treat the disorders of the mind, conditions that account for a large proportion of aggregate human suffering [59]. We need not try to hide the large amount we still do not know about the causes of mental illness behind metaphorical brain talk.
